# Genetic inhibition of RIPK3 ameliorates functional outcome in controlled cortical impact independent of necroptosis

**DOI:** 10.1038/s41419-021-04333-z

**Published:** 2021-11-09

**Authors:** Limin Wu, Joon Yong Chung, Tian Cao, Gina Jin, William J. Edmiston, Suzanne Hickman, Emily S. Levy, Jordyn A. Whalen, Eliza Sophie LaRovere Abrams, Alexei Degterev, Eng H. Lo, Lorenzo Tozzi, David L. Kaplan, Joseph El Khoury, Michael J. Whalen

**Affiliations:** 1 Department of Pediatrics, Massachusetts General Hospital, Harvard Medical School, Boston, MA 02114 USA; 2 Department of Neurology, West China Hospital, Sichuan University, 610041 Chengdu, Sichuan China; 3 Department of Medicine, Center for Immunology and Inflammatory Disease, Massachusetts General Hospital, Boston, USA; 4 Developmental, Molecular and Chemical Biology, Tufts University School of Medicine, Boston, MA USA; 5 Department of Radiology, Massachusetts General Hospital, Boston, MA 02115 USA; 6 Department of Neurology, Massachusetts General Hospital, Boston, MA 02115 USA; 7 Department of Biomedical Engineering, Tufts University, Medford, MA 02155 USA

**Keywords:** Molecular neuroscience, Brain injuries

## Abstract

Traumatic brain injury (TBI) is a leading cause of death and disability with no specific effective therapy, in part because disease driving mechanisms remain to be elucidated. Receptor interacting protein kinases (RIPKs) are serine/threonine kinases that assemble multi-molecular complexes that induce apoptosis, necroptosis, inflammasome and nuclear factor kappa B activation. Prior studies using pharmacological inhibitors implicated necroptosis in the pathogenesis of TBI and stroke, but these studies cannot be used to conclusively demonstrate a role for necroptosis because of the possibility of off target effects. Using a model of cerebral contusion and *RIPK3* and *mixed lineage kinase like* knockout (*MLKL*^−/−^) mice, we found evidence for activation of RIPK3 and MLKL and assembly of a RIPK1-RIPK3-MLKL necrosome complex in pericontusional brain tissue. Phosphorylated forms of RIPK3 and MLKL were detected in endothelium, CD11b + immune cells, and neurons, and RIPK3 was upregulated and activated in three-dimensional human endothelial cell cultures subjected to CCI. *RIPK3*^−/−^ and *MLKL*^−/−^ mice had reduced blood-brain barrier damage at 24 h (*p* < 0.05), but no differences in neuronal death (6 h, *p* = ns in CA1, CA3 and DG), brain edema (24 h, *p* = ns), or lesion size (4 weeks, *p* = ns) after CCI. *RIPK3*^−/−^, but not *MLKL*^−/−^ mice, were protected against postinjury motor and cognitive deficits at 1–4 weeks (*RIPK3*^−/−^ vs WT: *p* < 0.05 for group in wire grip, Morris water maze hidden platform trials, *p* < 0.05 for novel object recognition test, *p* < 0.01 for rotarod test). *RIPK3*^−/−^ mice had reduced infiltrating leukocytes (*p* < 0.05 vs WT in CD11b + cells, microglia and macrophages), HMGB1 release and interleukin-1 beta activation at 24–48 h (*p* < 0.01) after CCI. Our data indicate that RIPK3 contributes to functional outcome after cerebral contusion by mechanisms involving inflammation but independent of necroptosis.

## Introduction

Severe traumatic brain injury (TBI) is a leading cause of death in young people, accounting for over 250,000 hospitalizations and over 50,000 deaths/year in the United States [1], with total cost estimates for medical care, lost productivity and rehabilitation as high as $100 billion annually [2]. Treatment of patients with severe TBI is supportive and directed toward controlling intracranial hypertension [3]. Cerebral contusion is a TBI subtype featuring intraparenchymal hemorrhage, blood-brain barrier damage, brain edema, a robust inflammatory response, and programmed cell death. No specific therapy has been shown to improve neurological outcome in patients with contusion or any other type of TBI, in part because of incomplete understanding of the relevant disease mechanisms.

Receptor interacting protein kinase-3 (RIPK3) is a serine/threonine kinase best known for its role in programmed necrosis (“necroptosis”). When activated downstream of tumor necrosis factor receptors or toll-like receptors, RIPK3 dimerizes with RIPK1 via their RIP homotypic interaction motif (RHIM) domains, and RIPK1 phosphorylates and activates RIPK3 at Ser232. Under conditions of relative caspase-8 inhibition, recruitment of the mixed lineage kinase like protein (MLKL) assembles a RIPK1-RIPK3-MLKL necrosome complex, where phosphorylation of MLKL-Ser345 by RIPK3 induces MLKL polymers that disrupt the plasmalemma and execute necroptosis [4–6]. RIPK3 can also contribute to apoptosis via its scaffold function in a RIPK1-FADD-RIPK3-Caspase-8 ripoptosome complex [7]. Cell death-independent functions for RIPK3 have also recently been reported [8, 9]. RIPK3 induces inflammation via kinase-independent inflammasome activation (scaffold activity) [10–12] and via a kinase-dependent, necroptosis-independent pathway that activates NFkB and cFOS-mediated transcription via extracellular regulated kinase (ERK) signaling [13]. RIPK3 also mediates inflammation through CAP-dependent cytokine translation independently of necroptosis [14], and via release of damage-associated molecular patterns during necroptotic cell death. RIPK3 contributes to cell death and neurological dysfunction in Gaucher’s disease and stroke models [15, 16], and *RIPK3* knock out (*RIPK3*^*−/−*^) was associated with reduced post-injury cognitive dysfunction in a controlled cortical impact (CCI) model [17]. However, whether necroptosis drives clinically relevant outcomes in TBI models cannot be conclusively determined in *RIPK3*^−/−^ mice because of the necroptosis-independent functions of RIPK3.

Here, we tested the hypothesis that RIPK3 is a disease driver of functional outcome after TBI independent of necroptosis mechanisms, by comparing *RIPK3*^−/−^ to *MLKL*^−/−^ mice in a CCI model. Our secondary goal was to identify cell types that activate RIPK3 and MLKL early after CCI and identify potential mechanisms downstream of RIPK3 associated with cognitive impairment, with a focus on interleukin-1 beta (IL-1β) because of its known involvement in postinjury cognitive deficits in cerebral contusion models [18].

## Materials and methods

### Animals

Experiments were performed according to ARRIVE guidelines [19]. All experiments were performed by investigators blinded to study group and were approved by the Animal Experimentation Ethics Committee of Massachusetts General Hospital and complied with the NIH Guide for the Care and Use of Laboratory Animals. Mice were given free access to food and water and were housed in laminar flow racks in a temperature-controlled room with 12-h day/night cycles. Mice (males, 2–4 months of age) were randomized to sham and injury groups. *RIPK3*^−/−^ mice were generated at Genentech as previously described [20]. *MLKL*^−/−^ [21] mice were obtained from Dr. Siddharth Balachandran (Fox Chase Cancer Center, Philadelphia, PA). *MLKL*^−/−^ mice are derived solely from C57Bl/6J without passenger DNA from other strains, hence C57Bl/6J mice were controls for *MLKL*^−/−^. C57BL/6NJ mice were used as age matched controls for *RIPK3*^−/−^ mice except in behavior studies. For behavior experiments in *RIPK3*^−/−^ mice, C57Bl/6J mice were bred with *RIPK3*^−/−^ mice (C57Bl/6NJ background) and the F1 heterozygotes were interbred to generate *RIPK3*^*+/*+^ and *RIPK3*^−/−^ littermates on a mixed C57Bl/6J and C57Bl/6N background. This was done to control for genetic predisposition of the C57Bl/6NJ background to develop vision loss in adulthood. The genotype of all lines was confirmed by PCR with the suppliers’ protocol.

### Induction of controlled cortical impact

The mouse CCI model was used as previously described [22] except that a depth of 1.2 mm was used. Mice were induced with 4% isoflurane, 70% N_2_O and balance O_2_ and placed in a stereotactic frame. Anesthesia was titrated to quiet respirations and lack of toe pinch response at a level that avoids hypotension [23]. A 5-mm craniotomy was performed over the left parietotemporal cortex and the bone flap removed. CCI was produced using a pneumatic cylinder with a 3-mm flat-tip impounder, velocity 6.0 m/s, and depth of 1.2 mm. Sham-injured mice received craniotomy without CCI. Following sham injury or CCI, the bone flap was discarded and the scalp sutured closed. Mice were returned to their cages to recover from anesthesia.

### Behavioral testing

Behavior testing was performed during the same time of day (7 a.m. to 11:30 a.m.). Prior to each test, mice were acclimatized to the room for at least 30 min. To reduce variability, mice were pretrained in Morris water maze (MWM), novel object recognition, rotarod, and wire grip tests before injury. Wire grip was tested beginning on postinjury day 1 and all other tests were performed between 3 and 4 weeks after injury.

### Wire grip test

Gross vestibulomotor function was assessed using a wire grip test on post-injury days 1–60. The wire grip test consisted of placing the mouse on a wire (45 cm long) suspended between two poles 45 cm high, and grading the ability of mice to traverse the wire over 60 s [24].

### Rotarod

A well-established rotarod task (in which the speed was increased from 4 to 40 rotations per minute over 200 s was utilized to evaluate fine locomotor and balance function [25]. Mice were trained for three trials/day for 3 days to establish baseline performance. The test was ended if the mouse fell off the rod or completed two full revolutions. The average daily scores for each subject were used in the statistical analyses. Post-injury protocol: beginning on post-injury day 21, three daily trials were done for each mouse for a total of three days.

### Morris water maze

On each day of testing, mice were acclimatized to the room for at least 30 min. Mice were first tested in a MWM task at baseline before the injury and again in a reverse MWM paradigm at 3 weeks after injury. The MWM was performed as previously described with minor modifications [26]. Each mouse was subjected to 7 hidden platform trials (1–2 trials per day). Probe trials were performed 24 h after the last hidden platform trial by allowing the mice to swim in the tank for 30 s with the platform removed, and recording the time spent in the target quadrant. Finally, two visible platform trials with the platform raised 0.5 cm above the water and clearly marked with tape were performed.

### Novel object recognition test

Mice were placed in an arena (a white plastic box, 60 cm × 40 cm × 30 cm) with two identical objects for 10 min. Following a 24 h inter-trial interval, the mice were returned to the arena with one familiar object and one novel object for 5 min. The arena was cleaned with 70% ethanol between each mouse. The time spent interacting with each object was recorded by ANYMAZE software.

### Assessment of lesion volume

Morphometric image analysis was used to determine lesion volume at 6 weeks after CCI according to the method of Cavalieri as previously described [24]. Lesion volume was the difference between non-injured and injured hemispheric brain tissue volume and was expressed in mm^3^.

### Assessment of PI-positive and fluoro Jade B Cell counts

Propidium Iodide (PI; 10 mg/ml; Sigma, St Louis, MO, USA) diluted in phosphate-buffered saline (PBS) was administered intraperitoneally (1 mg/kg) in a total volume of not more than 200 μl 1 hour before killing. Mice were killed at 6 h after CCI, the brains were frozen in nitrogen vapor, and cryostat brain sections (20 μm) were cut at 250 μm intervals from anterior to posterior hippocampus. Cryostat sections were placed on poly-l-lysine slides and stored at −80 °C. For detection of PI-labeled cells, the brain sections were fixed in 100% ethanol for 10 min at room temperature, cover slipped, and photographed on a Nikon Eclipse T300 fluorescence microscope (Tokyo, Japan) using excitation/emission filters 568/585. PI-positive cells were quantitated in cortex and hippocampus in three brain sections separated by at least 150–200 μm as previously described [27]. Regions of interest were areas within the contusion as well as within the immediate peri-contusion zone.

Fluoro Jade B staining was done according to the manufacturer’s instructions using PBS-perfused, fresh frozen brain tissue sections. Positive staining was detected using fluorescence microscopy with excitation/emission filters 488/525 nm.

### Brain edema

Brains were removed at 24 h after CCI, bisected into left and right hemispheres, and each hemisphere was weighed (wet weight). Brains were then dried at 85 °C for 72 h, and dry weights were obtained. The percentage of brain water content was expressed as (wet-dry weight)/wet weight × 100%.

### Assessment of blood–brain barrier permeability

Evans Blue (5 ml/kg of a 2% solution) was injected intravenously 1 h after CCI and allowed to circulate for 23 h to account for dynamic, time dependent changes in BBB permeability during the entire measurement period [28]. At 24 h mice were transcardially perfused with PBS and brains were removed and placed in 3 ml *N*,*N*-dimethylformamide for 72 h at room temperature. Evans blue concentration was analyzed by spectrophotometry (585 nm) using known standards. Results were expressed as milligrams Evans blue/gram brain.

### Flow cytometric analysis

Mice were transcardially perfused with PBS and brains were removed and subjected to enzymatic digestion using the Neural Tissue Dissociation Kit (P) (Miltenyi Biotec, Auburn, CA). Myelin debris was removed with myelin removal beads II (Miltenyi) and cells were labeled with fluorescence-labeled CD11b (M1/70) (Biolegend, #101218), CD45 (30-F11) (BD # 553079), and Ly6G (Biolegend, #127618) antibodies. Microglia (CD11b^high^CD45^med^), macrophages (CD11b^high^CD45^high^Ly6G^low^), neutrophils (CD11b^high^CD45^high^Ly6G^high)^, and lymphocytes (CD11b^low^CD45^high^) were quantitated by FACS as previously described [29].

### Isolation of brain cells by magnetic beads

Mice were transcardially perfused with PBS and the brain was removed, digested using a Neural tissue dissociation kit (Miltenyi Biotec, Auburn, CA), and mechanically dissociated with a plastic pipette. After centrifugation at 1000 × *g* (7 min), the cell pellet was resuspended and incubated with myelin removal beads (Miltenyi Biotec) for 40 min on ice. After washing in PBS, Dynabeads (Thermo Fisher Scientific) conjugated to anti-CD31 (BD Pharmingen, #550274) or anti-CD11b (Biolegend, #127618) were added and a magnetic separator was used to recover the bead-bound cells. Unbound cells were incubated with anti-ACSA + beads (Miltenyi Biotec, #130-097-678) and separated using LS columns (Miltenyi Biotec). Neurons were isolated using a neuron isolation kit (Miltenyi Biotec). Isolated cells were frozen at −80 °C until processing for western blot. We previously reported on the purity of isolated cells by our immunopanning protocol [30].

### Necrosome Isolation Assay

Brain tissue or isolated brain cells were lysed in 1% Triton X-100 lysis buffer (150 mM NaCl, 20 mM Tris-Cl [pH 7.5], 1% Triton X-100, 1 mM EDTA, 3 mM NaF, 1 mM B-glycerophosphate, 1 mM Sodium Orthovanadate, 5 μM Idoacetamide [Cysteine protease inhibitor], 2 μM *N*-ethylmaleimide [Cysteine protease inhibitor], Phosphatase and Protease inhibitor cocktails (Thermo Scientific). After sonication, samples were centrifuged at 1000 × *g* for 10 min to precipitate the nuclear fraction, and the supernatant was transferred to a new tube and spun at 20,000 × *g* for 30 min at 4 °C. Supernatants were collected and diluted in 4X Laemmli buffer to prepare soluble fractions for western blot analysis. Pellets (insoluble fraction/Necrosome fraction) were washed once in a fresh aliquot of lysis buffer and spun at 20,000 × *g* for 15 min. Following wash, the supernatant was discarded and pellets were dissolved in lysis buffer containing 6 M urea. Samples were then added to 4x Laemmli buffer. Fractions from both compartments were subjected to gel electrophoresis and probed for RIPK1 or RIPK3 by western blot.

### Caspase 8 activity assay

Caspase-8 enzymatic activity was assessed in cortical brain tissue homogenates using a luminescence Caspase-Glo 8 Assay kit from Promega, Inc. according to the manufacturer’s instructions.

### Interleukin-1 beta ELISA

IL-1 beta was assessed in brain tissue homogenates by ELISA (R&D Systems) according to the manufacturer’s instructions.

### Western blot analyses

Brain tissue or isolated cells from a block of tissue from the ipsilateral hemisphere containing the entire cerebral contusion were homogenized in RIPA buffer (EMD Millipore) containing protease and phosphatase inhibitors (Thermo Fisher Scientific). Protein content was quantitated with a colorimetric assay (Bio-Rad, Richmond, CA, USA). Samples were denatured by adding SDS sample buffer and boiling for 5 min, and 30 μg of protein was loaded into each well. Proteins were transferred to polyvinylidene diflouride membranes (Millipore, Immobilon transfer membrane, Bedford, MA, USA). After blocking with 5% milk in 0.1% Tris-buffered saline Tween (TBS-T) (10 mM Tris-HCl pH 7.6, 150 mM NaCl, 0.1% Tween 20) for 1 h, membranes were incubated overnight at 4 °C with primary antibodies: anti-RIPK1 (1:1000, BD Biosciences, #610458), anti-RIPK3 (1:1000, Prosci, #2283), anti-MLKL (1:2000, Millipore, MABC604), anti-mouse pMLKL (Cell signaling Technology [CST], #37333S), anti- mouse pRIPK3 (1:1000, CST, #91702S), anti-human pRIPK3 (1:1000, AbCam, #ab209384), anti-human pMLKL (1:1000, CST, #91689S), anti-human p-RIPK1 (1:2000, CST, #44590S), anti-human RIPK3 (1:1000, abcam, # ab56164), anti-beta-actin (1:10000, CST, #5125S), anti-IL1beta (1:1000, abcam, #ab9722), anti-HMGB1 (1:1000, abcam, #ab79823), and cleaved caspase-8 (1:1000, CST, #9429S). After incubation with peroxide-conjugated secondary antibodies (1:5000, Cell signaling Technology, anti rabbit: #7074S, anti-mouse: #7076S, anti rat:#7077S), proteins were visualized with ECL (EMD Millipore) detection. Optical density of protein bands was assessed using ImageJ software and bands of interest were normalized to beta-actin.

### Immunoprecipitation

Brain homogenates from a block of tissue containing the entire cerebral contusion were lysed in RIPA buffer containing protease and phosphatase inhibitors (Sigma-Aldrich), followed by sonication and centrifugation at 15,000 × *g* for 30 min. Supernatants containing protein complexes were incubated with anti-RIPK3 (Prosci, #2283) or anti-RIPK1 (BD bioscience, #610458) antibodies conjugated to Magnetic Protein G Dynabeads (Thermo Fisher Scientific) at 4 °C overnight. Beads were washed three times with washing buffer and proteins were eluted in 1X loading buffer and processed/ for western blot.

### Human primary brain microvascular endothelial cell culture on silk scaffolds

Porous silk scaffolds were prepared as previously reported [31]. Human primary brain microvascular endothelial cells (hpBMEC) were purchased from Cell Systems (Kirkland, WA) and cultured according to the manufacturer’s protocol. The silk scaffolds were coated with 20 mg/mL fibronectin (Sigma Aldrich) for 1 h at 37 °C before cell seeding. The coating solution was removed by vacuum aspiration and scaffolds allowed to dry in the hood for 15 min. The scaffolds were individually transferred to 24-well, ultra-low attachment plates (Corning, Corning, NY) and 2.5 × 105 cells were seeded for each scaffold in 50 μl of media, allowing the silk sponges to fully absorb the cell suspension. The plates were then stored in the incubator for 30 min, to allow cell adhesion, prior to adding 1 ml media to each well (complete classic medium with serum and culture boost, Cell Systems, Inc.) to fully cover the scaffolds. Cell-seeded sponges were cultured for seven days to allow formation of a confluent endothelial layer covering the whole surface of the scaffolds, replacing the media every 3 days.

### 3D in vitro CCI model

Three-dimensional scaffolds of human brain endothelial cells were placed on a cell culture dish and subjected to CCI using the same apparatus used for in vivo CCI (6 m/s, 1.2 mm depth) or to sham injury (no impact) as previously described [32]. Cultures were then returned to media and incubated at 37 °C for 24 h.

### Immunostaining

Mice were anesthetized and transcardially perfused with 30 ml PBS followed by 4% paraformaldehyde (PFA). Brains were post fixed in 4% PFA for 24 h and cryoprotected in 30% sucrose for 24 h then refrigerated at 4 °C in sucrose. Serial 20 um coronal sections were cut on a cryostat (Leica) from the anterior frontal lobes through the posterior extent of the dorsal hippocampus. Every 10th section was collected and mounted on slides and kept at −80 °C. Slides were boiled in 95 °C in antigen retrieval solution (Sigma, Allentown, PA, USA) for 20 min. Manufacturer-supplied blocking buffer was used for each reaction. The sections were incubated with HMGB1 primary antibodies (1:1000, abcam, #ab79823) in 10% Normal Goat Serum in PBS overnight at 4 °C. After washing in PBS, slides were incubated in FITC-conjugated secondary antibodies (Invitrogen, #A11034) in PBS for 1 h at room temperature followed by streptavidin-labeled fluorophores. Labeled sections were visualized with a Nikon fluorescence Microscope.

### Collection of cerebrospinal fluid from mice

Mice were anesthetized with isoflurane and the head fixed in a stereotactic apparatus. the end of a pulled glass pipette was advanced into the cisterna magna with an occipital approach. Cerebrospinal fluid (CSF) was obtained by cisterna magna puncture. Only CSF that remained clear throughout the collection period was used for analyses. Approximately 1–4 μl CSF was typically obtained per mouse.

### Statistical analysis

Data are mean ± standard error of the mean (SEM). All data with *n* = 5 or more per group passed normality tests (Anderson-Darling test and others). Data with *n* = 4/group were densitometry or cell counts (continuous) and expected to be normally distributed. Data were analyzed using GraphPad PRISM VII (La Jolla, CA). MWM hidden and visible platform, rotarod and wire grip test data were analyzed by two-factor repeated measures analysis of variance (group × time). Other data were analyzed by unpaired *t*-test or one-way ANOVA depending on the number of groups. Significance was set at *p* < 0.05. Table 1 lists the statistical results for all comparisons. All statistical analyses were performed blinded to groups, except for western blot densitometry data.

**Table 1:** Statistics for the results.

Fig. 1		Time point	Sample size	Test	*P* value	*t*, df, or *F* (Dfn, Dfd)
B	Total-caspase8	24 h	*n* = 6–7	Unpaired *t* test, two tailed	0.363	*t* = 0.9487, df = 11
	43kd cleaved-caspase8	24 h	*n* = 6–7	Unpaired *t* test, two tailed	0.002	*t* = 4.172, df = 11
	18kd cleaved-caspase 8	24 h	*n* = 6–7	Unpaired *t* test, two tailed	0.000	*t* = 5.624, df = 11
C	Caspase 8 activity	24 h	*n* = 7–8	Unpaired *t* test, two tailed	<0.0001	*t* = 6.76, df = 13
E	RIPK1	Sham/3 h/6 h/24 h		One-way anova	0.9953	*F* (3, 16) = 0.02219
		Sham vs. 3 h	*n* = 3–7	Dunnett test	0.9979	
		Sham vs. 6 h	*n* = 3–7	Dunnett test	0.9987	
		Sham vs. 24 h	*n* = 7	Dunnett test	0.9993	
F	RIPK3	Sham/3 h/6 h/24 h		One-way anova	0.0121	*F* (3, 16) = 5.033
		Sham vs. 3 h	*n* = 3–7	Dunnett test	0.0334	
		Sham vs. 6 h	*n* = 3–7	Dunnett test	0.0482	
G		Sham vs. 24 h	*n* = 7	Dunnett test	0.0129	
	MLKL	Sham/3 h/6 h/24 h		One-way anova	0.0004	*F* (3, 17) = 10.55
		Sham vs. 3 h	*n* = 4–8	Dunnett test	0.0391	
		Sham vs. 6 h	*n* = 3–8	Dunnett test	0.2926	
		Sham vs. 24 h	*n* = 6–8	Dunnett test	0.0001	
I	RIPK1 expression in CD31 (WT)	24 h	*n* = 7	Unpaired *t* test, two tailed	0.027	*t* = 2.512 df = 12
	RIPK1 expression in CD11b	24 h	*n* = 5–6	Unpaired *t* test, two tailed	0.022	*t* = 2.76, df = 9
	RIPK1 expression in neuron	24 h	*n* = 5–6	Unpaired *t* test, two tailed	0.010	*t* = 3.28, df = 9
J	RIPK3 expression in CD31	24 h	*n* = 6	Unpaired *t* test, two tailed	0.009	*t* = 3.224, df = 10
	RIPK3 expression in CD11b	24 h	*n* = 5	Unpaired *t* test, two tailed	0.083	*t* = 1.982, df = 8
	RIPK3 expression in neuron	24 h	*n* = 5	Unpaired *t* test, two tailed	0.370	*t* = 0.95, df = 8
K	MLKL epxression in CD31_	24 h	*n* = 6	Unpaired *t* test, two tailed	0.924	*t* = 0.09827, df = 10
	MLKL expression in CD11b	24 h	*n* = 6	Unpaired *t* test, two tailed	0.568	*t* = 0.5903, df = 10
	MLKL expression in neuron	24 h	*n* = 5	Unpaired *t* test, two tailed	0.030	*t* = 2.636, df = 8

Fig. 2		Time point	Sample size	Test	*P* value	*t*, df/F(DFn,DFd)
B	RIPK1 in triton fraction	Sham 3 h 24 h		One-way anova	0.270	*F* (2, 6) = 1.642
		Sham vs 3 h	*n* = 3	Dunnett test	0.2736	
		Sham vs 24 h	*n* = 3	Dunnett test	0.2725	
	RIPK1 in urea fraction	Sham 3 h 24 h		One-way anova	0.0071	*F* (2, 6) = 12.60
		3 h	*n* = 3	Dunnett test	0.2786	
		24 h	*n* = 3	Dunnett test	0.0048	
C	RIPK3 in trition fraction	Sham 3 h 24 h	*n* = 3	One-way anova	0.0315	*F* (2, 6) = 6.503
		3 h	*n* = 3	Dunnett test	0.0874	
		24 h	*n* = 3	Dunnett test	0.0221	
D	MLKL in triton fraction	Sham 3 h 24 h	*n* = 3	One-way anova	0.1161	*F* (2, 6) = 3.149
		3 h	*n* = 3	Dunnett test	0.3572	
		24 h	*n* = 3	Dunnett test	0.0794	
	MLKL in urea fraction	Sham 3 h 24 h	*n* = 3	One-way anova	0.019	*F* (2, 6) = 8.244
		3 h	*n* = 3	Dunnett test	0.187	
		24 h	*n* = 3	Dunnett test	0.0119	
F	p-RIPK3 in CD11b	3 h	*n* = 3	Unpaired *t* test, two tailed	0.011	*t* = 4.536, df = 4
	p-RIPK3 in neuron	3 h	*n* = 4	Unpaired *t* test, two tailed	<0.0001	*t* = 10.25, df = 6
	p-MLKL in neuron	3 h	*n* = 3	Unpaired *t* test, two tailed	0.041	*t* = 2.973, df = 4
H	p-RIPK3 in CD31	24 h	*n* = 3	Unpaired *t* test, two tailed	0.011	*t* = 4.485, df = 4
	p-RIPK3 in CD11b	24 h	*n* = 3	Unpaired *t* test, two tailed	0.044	*t* = 2.910, df = 4
	p-RIPK3 in neuron	24 h	*n* = 4	Unpaired *t* test, two tailed	0.045	*t* = 2.520, df = 6
	p-MLKL in neuron	24 h	*n* = 4	Unpaired *t* test, two tailed	0.023	*t* = 3.041, df = 6
K	p-RIPK3 3D culture	24 h	*n* = 6	Unpaired *t* test, two tailed	0.003	*t* = 3.828, df = 10
	RIPK3 3D culture	24 h	*n* = 3	Unpaired *t* test, two tailed	0.046	*t* = 2.852, df = 4

Fig. 3		Comparison	Sample size	Test	Column factor		Time x column factor		Time		Subject	
B	RIPK3KO wire grip		*n* = 19–21	Two-way RM ANOVA	0.019	*F* (1, 38) = 6.033	0.140	*F* (8, 304) = 1.549	<0.0001	*F* (4.535, 172.3) = 85.86	<0.0001	*F* (38, 304) = 2.681
C	RIPK3KO rotarod	Pre WT vs RIPK3KO	*n* = 9–10	Unpaired *t* test, two tailed	0.545	*t* = 0.6177, df = 17						
		Post WT vs RIPK3KO	*n* = 9–11	Two-way RM ANOVA	0.017	*F* (1, 17) = 6.961	0.876	*F* (3, 51) = 0.2291	<0.0001	*F* (1.892, 32.16) = 26.30	0.279	F [17, 51] = 1.226
D	RIPK3KO mwm	Hidden platform	*n* = 9–12	Two-way RM ANOVA	0.028	*F* (1, 19) = 5.667	0.279	*F* (6, 114) = 1.266	<0.0001	*F* (4.105, 77.99) = 8.564	<0.0001	*F* (19, 114) = 5.452
		Visible platform	*n* = 9–10	Two-way RM ANOVA	0.253	*F* (1, 17) = 1.399	0.000	F [1, 17] = 20.55	0.632	F [1, 17] = 0.2385	0.164	F [17, 17] = 1.623
E	RIPK3KO probe test	WT pre vs WT post	*n* = 9	Unpaired *t* test, two tailed	0.005	*t* = 3.220, df = 16						
		RIPK3−/− pre vs post	*n* = 12	Unpaired *t* test, two tailed	0.680	*t* = 0.4176, df = 22						
F	RIPK3KO NORT	WT pre old vs novel	*n* = 10	Paired *t* test	0.001	*t* = 4.852, df = 9						
		WT post old vs novel	*n* = 10	Paired *t* test	0.615	*t* = 0.5203, df = 9						
		RIPK3KO pre old vs novel	*n* = 9	Paired *t* test	0.001	*t* = 5.464, df = 8						
		RIPK3KO post old vs novel	*n* = 9	Paired *t* test	0.033	*t* = 2.565, df = 8						
G	MLKLKO wire grip	Wire grip	*n* = 14–16	Two-way RM ANOVA	0.314	*F* (1, 28) = 1.051	0.237	*F* (6, 152) = 1.354	<0.0001	*F* (4.133, 104.7) = 32.89		
H	MLKL KO rotarod	WT pre vs MLKL pre	*n* = 12–13	Unpaired *t* test, two tailed	0.969	*t* = 0.03919, df = 23						
		WT post vs MLKL post	*n* = 12–13	Two-way RM ANOVA	0.700	*F* (1, 23) = 0.1523	0.972	*F* (3, 69) = 0.07760	0.131	*F* (1.597, 36.73) = 2.238	<0.0001	F [23, 69] = 3.342
I	MLKLKO MWM	Hidden platform	*n* = 14–15	Two-way RM ANOVA	0.321	*F* (1, 27) = 1.020	0.079	*F* (6, 162) = 1.931	<0.0001	*F* (3.562, 96.19) = 7.131	<0.0001	*F* (27, 162) = 7.589
		Visible platform	*n* = 14–15	Two-way RM ANOVA	0.003	*F* (1, 27) = 11.08	0.253	F (1, 27) = 1.366	0.573	F [1, 27] = 0.3257	0.144	F [27, 27] = 1.514
J	MLKLKO probe test	WT pre vs WT post	*n* = 14–16	Unpaired *t* test, two tailed	0.001	*t* = 3.948, df = 29						
		MLKL KO pre vs post CCI	*n* = 14–17	Unpaired *t* test, two tailed	0.049	*t* = 2.062, df = 27						
		WT post CCI vs MLKL post CCI	*n* = 14–18	Unpaired *t* test, two tailed	0.408	*t* = 0.8410, df = 27						

Fig. 4		Region	Sample size	Test	*p* value	*t*, df
B	RIPK3KO vs WT PI	CA1	*n* = 8	Unpaired *t* test, two tailed	0.725	*t* = 0.3591, df = 14
		CA3	*n* = 8	Unpaired *t* test, two tailed	0.562	*t* = 0.5943, df = 14
		DG	*n* = 8	Unpaired *t* test, two tailed	0.090	*t* = 1.820, df = 14
		Cortex	*n* = 8	Unpaired *t* test, two tailed	0.721	*t* = 0.3641, df = 14
D	FJB + cell in WT vs RIPK3KO	CA1	*n* = 6	Unpaired *t* test, two tailed	0.213	*t* = 1.331, df = 10
		CA3	*n* = 6	Unpaired *t* test, two tailed	0.775	*t* = 0.2943, df = 10
		DG	*n* = 6	Unpaired *t* test, two tailed	0.187	*t* = 1.418, df = 10
		Cortex middle	*n* = 6	Unpaired *t* test, two tailed	0.504	*t* = 0.6931, df = 10
		Cortex lateral	*n* = 6	Unpaired *t* test, two tailed	0.979	*t* = 0.02650, df = 10
F	WT vs RIPK3−/− lesion volume		*n* = 9–10	Unpaired *t* test, two tailed	0.632	*t* = 0.4872, df = 17
H	WT vs MLKL−/− lesion volume		*n* = 14–16	Unpaired *t* test, two tailed	0.436	*t* = 0.7903, df = 28

Fig. 5					Ipsilateral		Contralateral				WT con vs ipsi	KO con vs ipsi
		Time point	Sample size	Test	*p* value	*t*, df	*p* value	*t*, df			*p* value	*p* value
B	RIPK3 vs WT evansblue	24 h	*n* = 7	Unpaired *t* test, two tailed	0.044	*t* = 2.250, df = 12	0.601	*t* = 0.5375, df = 12	0.022		2.522E-07	8.856E-05
D	WT vs MLKL/-/ evans blue	24 h	*n* = 6	Unpaired *t* test, two tailed	0.027	*t* = 2.580, df = 10	0.260	*t* = 1.195, df = 10	0.021		4.142E-06	6.657E-05
E	WT vs RIPK3-/- edema	24 h	*n* = 4–5	Unpaired *t* test, two tailed	0.162	*t* = 1.565, df = 7	0.484	*t* = 0.7387, df = 7	0.157	*t* = 1.583, df = 7	6.696E-06	1.066E-04
F	WT vs MLKL edema	24 h	*n* = 6	Unpaired *t* test, two tailed	0.095	*t* = 1.847, df = 10	0.026	*t* = 2.613, df = 10	0.586	*t* = 0.5625, df = 10	3.087E-05	3.678E-02

Fig. 6		Comparison	Sample size	Test	*p* value	*t*, df
B	CD11b 48 h	Sham WT vs RIPK3KO	*n* = 4	Unpaired *t* test, two tailed	0.529	*t* = 0.6678, df = 6
		CCI WT vs RIPK3KO	*n* = 6	Unpaired *t* test, two tailed	0.016	*t* = 2.879, df = 10
C	Microglia 48 h	Sham WT vs RIPK3KO	*n* = 4	Unpaired *t* test, two tailed	0.604	*t* = 0.5477, df = 6
		CCI WT vs RIPK3KO	*n* = 6	Unpaired *t* test, two tailed	0.027	*t* = 2.595, df = 10
D	Macrophage 48 h	Sham WT vs RIPK3KO	*n* = 4	Unpaired *t* test, two tailed	0.910	*t* = 0.1177, df = 6
		CCI WT vs RIPK3KO	*n* = 6	Unpaired *t* test, two tailed	0.013	*t* = 3.028, df = 10
E	Neutrophil 48 h	Sham WT vs RIPK3KO	*n* = 4	Unpaired *t* test, two tailed	0.529	*t* = 0.6675, df = 6
		CCI WT vs RIPK3KO	*n* = 6	Unpaired *t* test, two tailed	0.221	*t* = 1.307, df = 10
F	Lymphocytes 48 h	Sham WT vs RIPK3KO	*n* = 4	Unpaired *t* test, two tailed	0.581	*t* = 0.5833, df = 6
		CCI WT vs RIPK3KO	*n* = 6	Unpaired *t* test, two tailed	0.443	*t* = 0.7990, df = 10
G	IL1b elisa brain tissue	Sham WT vs RIPK3KO	*n* = 5–6	Unpaired *t* test, two tailed	0.007	*t* = 3.451, df = 9
		CCI WT vs RIPK3KO	*n* = 6–9	Unpaired *t* test, two tailed	0.002	*t* = 3.978, df = 13
I	CD11b + 3 w	WT vs RIPK3KO	*n* = 5–6	Unpaired *t* test, two tailed	0.042	*t* = 2.376, df = 9
J	Microglia 3 w	WT vs RIPK3KO	*n* = 5–6	Unpaired *t* test, two tailed	0.037	*t* = 2.450, df = 9
K	Macrophage 3 w	WT vs RIPK3KO	*n* = 5–6	Unpaired *t* test, two tailed	0.628	*t* = 0.5014, df = 9
L	Lymphocytes 3 w	WT vs RIPK3KO	*n* = 5–6	Unpaired *t* test, two tailed	0.971	*t* = 0.03764, df = 9

Fig. 7		Time point	Sample size	Test	*p* value	*t*, df
C	Neurons	24 h	*n* = 3	Unpaired *t* test, two tailed	0.011	*t* = 4.436, df = 4
E	WT	24 h	*n* = 5	Unpaired *t* test, two tailed	0.006	*t* = 5.409, df = 4
	RIPK3−/−	24 h	*n* = 4	Unpaired *t* test, two tailed	0.448	*t* = 0.8714, df = 3
	MLKL	24 h	*n* = 3	Unpaired *t* test, two tailed	<0.0001	*t* = 105.7, df = 2

Supplement Fig. 2						
B	p-RIPK1 sham vs CCI	24 h	*n* = 6	Unpaired *t* test, two tailed	0.525	*t* = 0.6587,df = 10
	RIPK1 sham vs CCI	24 h	*n* = 6	Unpaired *t* test, two tailed	0.368	*t* = 0.9419,df = 10
C	p-MLKL sham vs CCI	24 h	*n* = 6	Unpaired *t* test, two tailed	0.306	*t* = 1.079,df = 10
	MLKL sham vs CCI	24 h	*n* = 6	Unpaired *t* test, two tailed	0.586	*t* = 0.5626, df = 10

Supplement Fig. 3		Sample size	Test	*p* value		Time x column factor		*t*, df
A	RIPK3KO vs WT baseline mwm	*n* = 9–12	Two-way RM ANOVA	0.468	*F* (1, 19) = 0.5478	0.966	*F* (6, 114) = 0.2314	
B	MLKLKO vs WT baseline mwm	*n* = 15–16	Two-way RM ANOVA	0.823	*F* (1, 29) = 0.05105	0.909	*F* (6, 174) = 0.3505	
C	RIPK3KO vs WT platform crossing	*n* = 9–12	Unpaired *t* test, two tailed	0.282				*t* = 1.106, df = 19
D	MLKLKO vs WT platform crossing	*n* = 15–16	Unpaired *t* test, two tailed	0.820				*t* = 0.2296, df = 27
E	RIPK3KO vs WT post CCI swim speed	*n* = 9–12	Two-way RM ANOVA	0.207	*F* (1, 19) = 1.709	*P* = 0.0921	*F* (6, 114) = 1.869	
F	MLKLKO vs WT post CCI swim speed	*n* = 14–15	Two-way RM ANOVA	0.267	*F* (1, 27) = 1.285	0.766	*F* (6, 162) = 0.5546	

## Results

### Caspase-8 is inactivated in injured brain after CCI

**Fig. 1:**
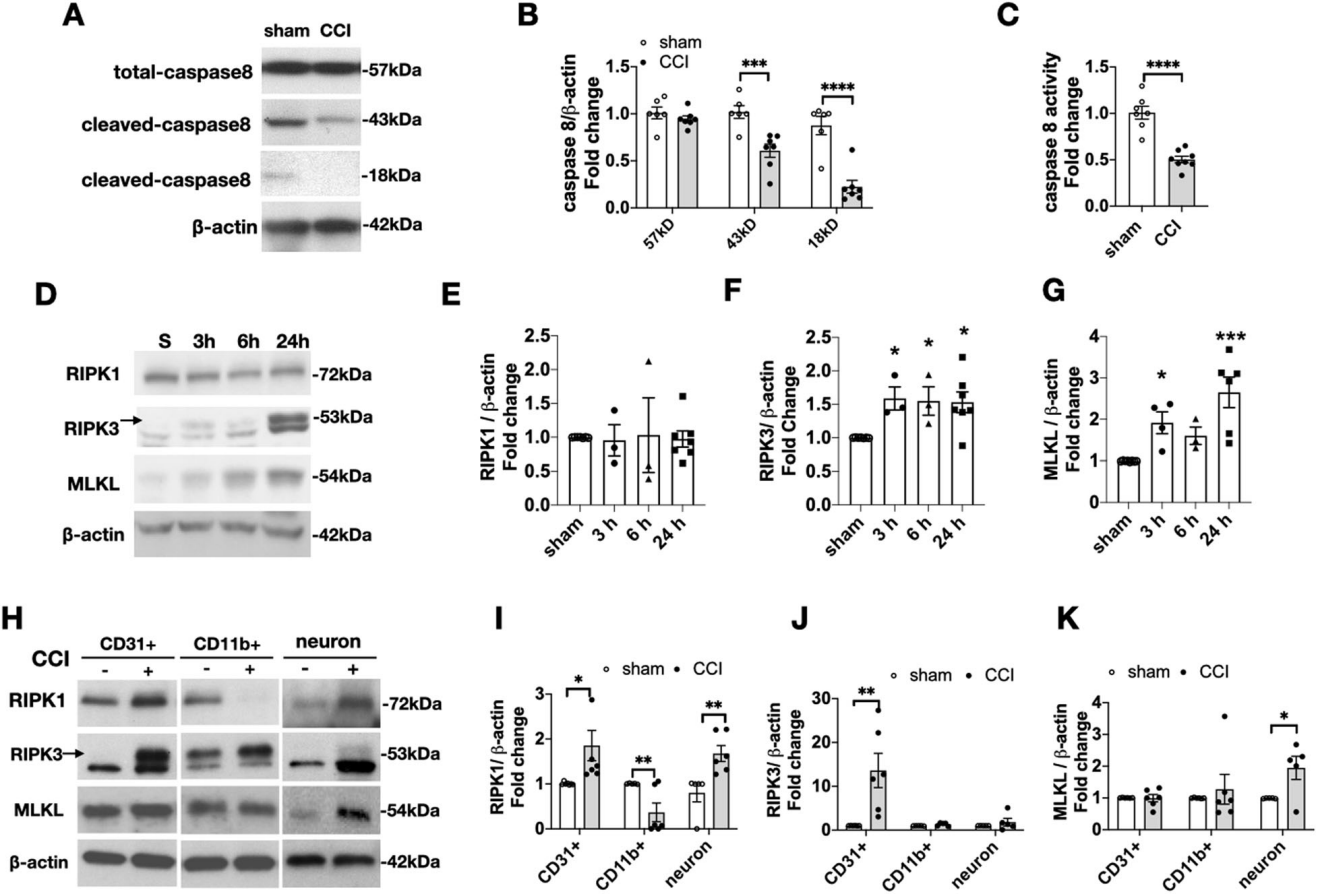
Time dependent changes in expression of caspase-8 and necroptosis proteins after CCI. **A** Representative western blot and **B** densitometry analyses of brain lysates from sham and injured mice showing reduction of cleaved caspase-8 43 kDa and 18 kDa bands (*n* = 6–7/group, **p = 0.002, ***p = 0.0002). **C** Caspase-8 enzymatic activity, assessed by conversion of a luminescent substrate, was decreased in brain homogenates from CCI compared to sham mice (*n* = 7–8/group, ****p < 0.0001, *t*-test). **D** Representative western blots and **E–G** densitometric analyses of RIPK1, RIPK3, and MLKL expression in sham and injured mouse brain showing increased RIPK3 and MLKL expression at 3–24 h after CCI. (*n* = 3–8 /group, *p < 0.05, **p < 0.01, ***p < 0.001). **H** Representative western blots and **I**–**K** densitometric analyses of RIPK1, RIPK3, and MLKL expression in immunopanned CD31 + endothelial cells, CD11b + cells and neurons isolated from sham and injured mouse brain at 24 h after CCI. RIPK1 was decreased in CD11b + but increased in endothelium and neurons; RIPK3 was increased only in endothelial cells and MLKL expression was increased in neurons (*n* = 5–6/group, *p < 0.05, **p < 0.01).

In human neurodegenerative diseases, inhibition of caspase-8 favors necroptosis by preventing cleavage and inactivation of RIPK1 [33]. At 24 h after CCI, expression of the caspase-8 cleavage product 43 kDa band was reduced by ~50% and the active fragment 18 kDa band by ~70% in injured cortical/hippocampal brain homogenates vs. sham (Fig. 1A, B). Moreover, capase-8 activity was also decreased by ~50% in brain homogenates from injured mice vs. sham (Fig. 1C). Thus, caspase-8 protein expression and activity are reduced after CCI, favoring conditions for necroptosis.

### RIPK1, RIPK3, and MLKL are differentially expressed in contused brain after CCI

In whole brain homogenates, RIPK1 expression was not different in sham and CCI mice in the first 24 h after injury (Fig. 1D, E). RIPK3 was significantly increased by 24 hours (Fig. 1D, F), and MLKL was increased between 3 and 24 h vs. sham (Fig. 1D, G). RIPK3 was not detected in *RIPK3*^−/−^ and MLKL was not detected in *MLKL*^−/−^ mice; RIPK1 was not detected after RIPK1 shRNA treatment in BV2 cells, confirming the specificity of the antibodies used (Supplemental Fig. 1A, D).

### Cell-specific RIPK1, RIPK3, and MLKL expression after CCI

We next isolated brain cell populations by immunopanning and determined RIPK/MLKL expression by western blot (Fig. 1H–K). In sham injured mice, RIPK1 was detected in all cell types analyzed. At 24 h after CCI, RIPK1 was decreased by ~65% in CD11b + cells but was increased in neurons by 1.7-fold, and endothelium by 1.9-fold compared to sham (Fig. 1I). In contrast, RIPK3 expression was increased at 24 h after CCI in CD31 + endothelial cells by 13.6-fold but was not different vs. sham in CD11b + cells or neurons (Fig. 1H, J). MLKL expression was detectable in endothelium, CD11b + cells, and neurons of sham injured mice and was modestly increased after CCI in neurons by 2.0-fold (Fig. 1H, K). Of note, RIPK1, RIPK3, and MLKL were each detected in CCI microglia and macrophages sorted by FACS (Supplemental Fig. 1E, F).

### Activation of RIPK1, RIPK3, and MLKL and assembly of a necrosome complex after CCI

**Fig. 2:**
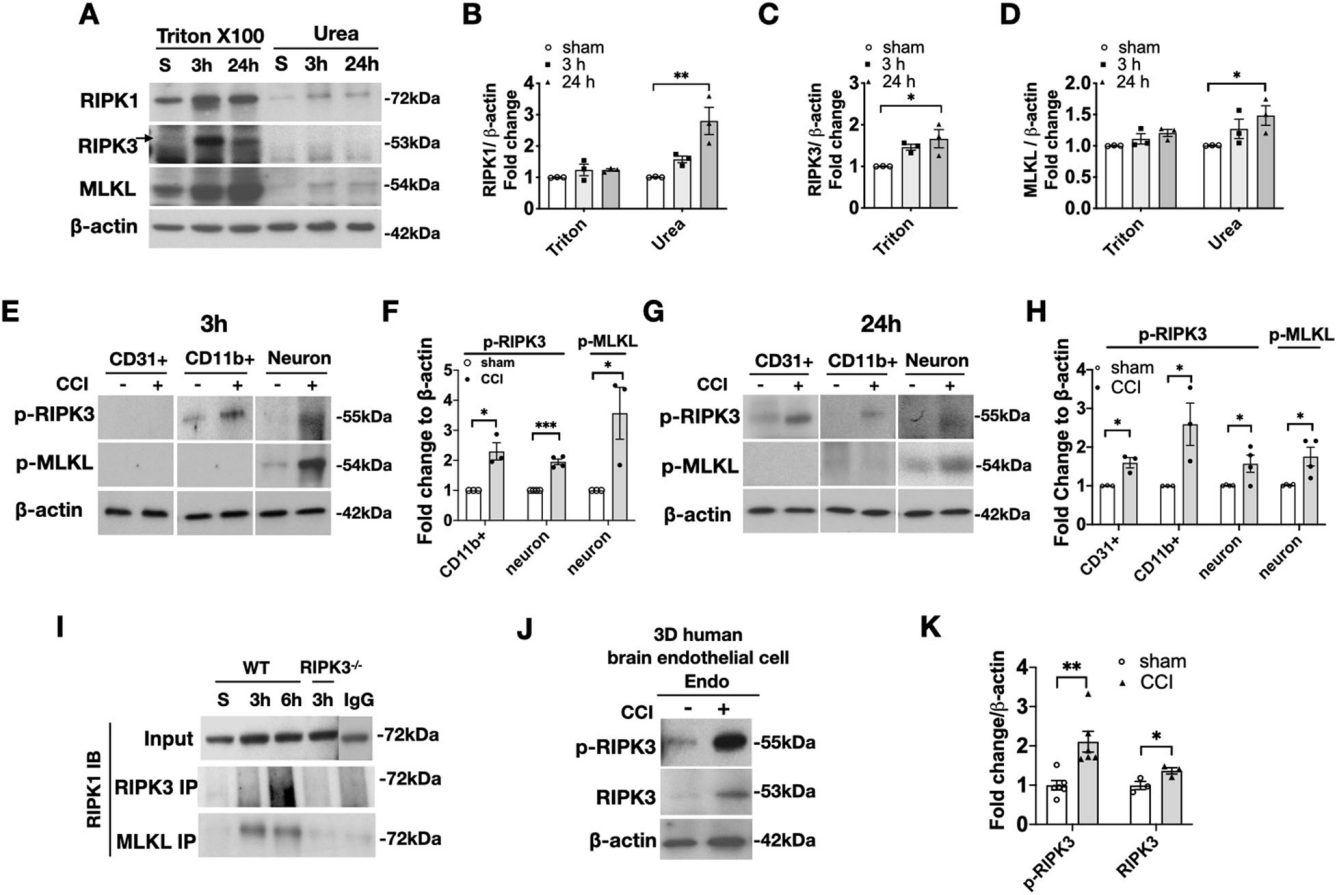
Evidence for activation of RIPK1, RIPK3, and MLKL in brain tissue and immunopanned cells after CCI. Mice were subjected to sham or CCI and brain tissue or cell populations isolated by immunopanning were subjected to immunoprecipitation and western blot analyses. **A** Representative western blot and **B–D** densitometry analyses of the 1% triton X100-soluble and 8 M urea-soluble brain tissue fractions from sham and CCI mice. RIPK1, RIPK3, and MLKL protein levels were increased by 24 h after CCI (*p < 0.05, **p < 0.01, *n* = 3/group). **E** Representative western blots and **F** densitometry analyses of phospho-RIPK3 (p-RIPK3) and p-MLKL expression in immunopanned CD31 + endothelial cells, CD11b + immune cells, and neurons isolated from sham and injured mouse brain at 3 h after CCI. p-RIPK3 was increased in CD11b + cells and neurons, p-MLKL was increased in neurons after CCI (*n* = 3–4/group, *p < 0.05, **p < 0.01). **G** Representative western blots and **H** densitometry analyses of phospho-RIPK3 (p-RIPK3) and p-MLKL expression in immunopanned CD31 + endothelial cells, CD11b + immune cells and neurons isolated from sham and injured mouse brain at 24 h after CCI. p-RIPK3 was increased in CD31 + cells, CD11b + cells and neurons, p-MLKL was increased in neurons after CCI (*n* = 3–4/group, *p < 0.05). **I** Representative immunoprecipitation (IP) with RIPK3 or MLKL pull down and western blot analyses for RIPK1 showing RIPK1-RIPK3-MLKL interaction early after CCI. Note lack of reactivity in *RIPK3*^*−/−*^ and with isotype control IgG. Data are representative of three independent experiments. **J** Representative western blots and **K** densitometric analyses of three-dimensional cultures of human brain endothelial cells subjected to sham or CCI showing increased expression of pRIPK3 and total RIPK3 at 24 h (*n* = 3–6/group, *p < 0.05, **p < 0.01).

Following activation, RIPK1 and RIPK3 may assemble necrosome complexes with MLKL with amyloid conformations that are insoluble in mild detergents such as Triton-X100 but soluble in urea [34]. In western blot analyses of contused brain tissue, RIPK1 expression was significantly increased in the triton-X100 insoluble (8 M urea soluble) cell fractions by 24 h (2.8-fold vs. sham), and MLKL expression was increased 1.5-fold, providing evidence for activation of RIPK1 and MLKL (Fig. 2A, B, D). In contrast, increased RIPK3 was detected in the triton-X100 soluble fraction at 3 (1.5-fold) and 24 h (1.7-fold) but was not detected in the urea fraction at either time point (Fig. 2A, C).

We next examined phosphorylated (activated) forms of RIPK3 and MLKL using antibodies that recognize specific phosphorylation events (RIPK3 at Thr231/ser232 and MLKL at Ser345) [4, 33, 35]. We previously reported that naïve (uninjured) mouse brain cells do not show phosphorylated forms of MLKL [30], and Supplemental Fig. 1G shows that pRIPK3 and pRIPK1 antibodies also do not react with immunopanned endothelium, immune cells, or neurons from naïve mouse brain, demonstrating that the immunopanning protocol itself does not lead to non-specific RIPK/MLKL activation. Compared to sham, at 3 h after CCI, pRIPK3 was induced in CD11b + cells (2.3-fold increase) and in neurons (2.0-fold) but was not detected in CD31 + endothelium from injured mice (Fig. 2E). At 24 h, increased pRIPK3 was detected in CD31 + endothelium (1.6-fold), in CD11b + cells (2.6-fold,), and in neurons (1.6-fold) (Fig. 2G, H). At 3 and 24 h, pMLKL was induced in neurons (3.6-fold increase at 3 h, 1.7-fold increase at 24 h) but was not detected in CD11b + or CD31 + cells (Fig. 2E–H). pMLKL was not detected in neurons from injured *RIPK3*^−/−^ mice, consistent with a requirement for RIPK3 in MLKL phosphorylation and necroptosis (Supplemental Fig. 1E). p-RIPK3 and p-MLKL were not detected in respective knockout tissue after CCI (Supplemental Fig. 1B, C). We did not detect pRIPK1 immunoreactivity in any of the immunopanned brain cell types at 24 h after CCI (data not shown).

Immunoprecipitation analyses provided further evidence of RIPK1-RIPK3-MLKL activation as pull down with RIPK3 and MLKL antibodies showed interaction of these proteins with RIPK1 at 3–6 h after CCI (Fig. 2I), suggesting assembly of a necrosome complex. Specificity for IP was confirmed by lack of detection of immunoprecipitants using control IgG as the IP reagent, and lack of RIPK1-RIPK3 interaction in *RIPK3*^−/−^ mouse brain (Fig. 2I). No increase in RIPK1-RIPK3 and RIPK1-MLKL interaction was detected in CCI vs. sham brain homogenates at 24 or 48 h (Fig. 2I and Supplemental Fig. 1H, 24 h data not shown).

We used three-dimensional human endothelial cell cultures to assess cell autonomous responses to TBI in vitro and draw parallels to the human condition [36]. We subjected human brain endothelial cells in three-dimensional silk scaffold cultures to trauma using the same CCI device and injury parameters as in vivo [32]. In human endothelial cells, significant induction of both total (1.4-fold) and phospho-RIPK3 (2.1-fold) was observed at 24 h in injured vs. sham injured cultures (Fig. 2J, K), with no difference in total and phospho-RIPK1 and MLKL (Supplemental Fig. 2A–C).

### RIPK3 deletion protects against neurological deficits after CCI

**Fig. 3:**
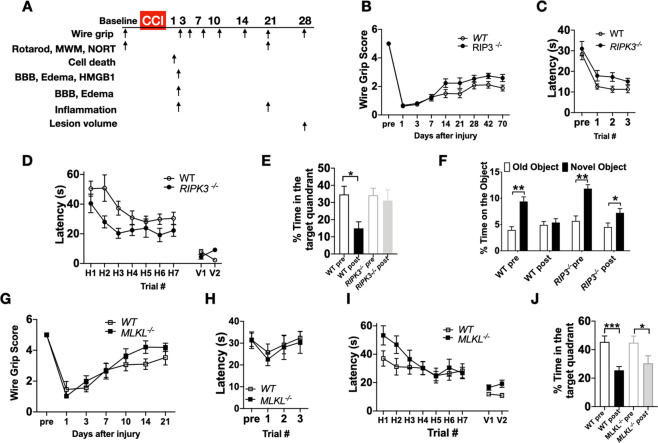
Effect of genetic inhibition of RIPK3 and MLKL on behavioral outcomes after CCI. **A** Schematic drawing of the experiments. After obtaining baseline behavioral data, mice were subjected to CCI and tested on the wire grip beginning on postinjury day one and up to the indicated times. Morris water maze (MMW), rotarod, and novel object recognition test (NORT) were performed beginning 3 weeks after injury. **B–F** *RIPK3*^*−/−*^ mice had significantly improved outcome after CCI vs. *WT* in tests of **B** wire grip (*n* = 19–21/group; *p < 0.05 for group, RM ANOVA), **C** rotarod (*n* = 9–12/group; *p < 0.05 for group, RM ANOVA), **D** MWM hidden platform trials (*n* = 9–12/group^−^; *p < 0.05 for group, RM ANOVA), **E** probe trials (*n* = 9-12/group, **p < 0.01) and **F** NORT (*n* = 9–10/group; *p < 0.05,**p < 0.01). **G–J** *MLKL*^*−/−*^ mice performed similarly to *WT* after CCI in **G** wire grip test (*n* = 14-16/group), **H** rotarod test (*n* = 12–13/group), **I** MWM hidden platform trials (*n* = 14–16/group, *p* = ns for group, RM ANOVA), and **J** probe trials (*n* = 14–16/group, *p < 0.05, ***p < 0.001).

We next sought to assess the possible functional significance of RIPK1/3 and MLKL in the CCI model. *RIPK3*^*+/+*^ and *RIPK3*^−/−^ mixed background (C57Bl/6N/C57Bl/6J) littermates performed similarly at baseline in wire grip, rotarod, MWM, and NORT tests. Following CCI, injured *RIPK3*^−/−^ littermates had modest but significantly improved performance vs. RIPK3^+/+^ in the wire grip (Fig. 3B) and rotarod tests (Fig. 3C). In MWM hidden platform trials, injured *RIPK3*^−/−^ performed significantly better than injured *RIPK3*^*+/+*^ after CCI with no difference in swim speeds between groups (Fig. 3D, Supplemental Fig. 3A, E). *RIPK3*^−/−^ mice had similar performance in probe trials pre- and post-CCI whereas *RIPK3*^*+/+*^ performed significantly worse after CCI vs. pre-injury (Fig. 3E). However, no differences between groups were observed in the number of platform crossings (Supplemental Fig. 3C). To confirm a possible role for RIPK3 in hippocampus-dependent memory post CCI, we performed a NORT in addition to probe trials. At baseline, *RIPK3*^*+/+*^ and *RIPK3*^−/−^ mice demonstrated preference for the novel object. After CCI, *RIPK3*^*+/+*^ demonstrated no preference for the novel object whereas injured *RIPK3*^−/−^ still maintained preference for the novel object (Fig. 3F), demonstrating a role for RIPK3 in hippocampal-dependent cognitive outcome after CCI.

*WT* and *MLKL*^−/−^ mice performed similarly at baseline in wire grip, rotarod, and MWM tests. Following CCI, *MLKL*^−/−^ mice had similar wire grip (Fig. 3G) and rotarod performance (Fig. 3H) vs. WT. Injured *MLKL*^−/−^ and *WT* mice performed similarly in MWM hidden platform trials but *MLKL*^−/−^ mice performed worse compared to *WT* in visible platform trials (Fig. 3I) with no differences in swim speed (Supplemental Fig. 3F). In probe trials *WT* and *MLKL*^−/−^ each performed at chance levels following CCI (Fig. 3J), and no differences between groups were observed in the number of platform crossings (Supplemental Fig. 3D).

### No difference in neuronal cell death or lesion volume after CCI in *RIPK3*^−/−^ and *MLKL*^−/−^ mice

**Fig. 4:**
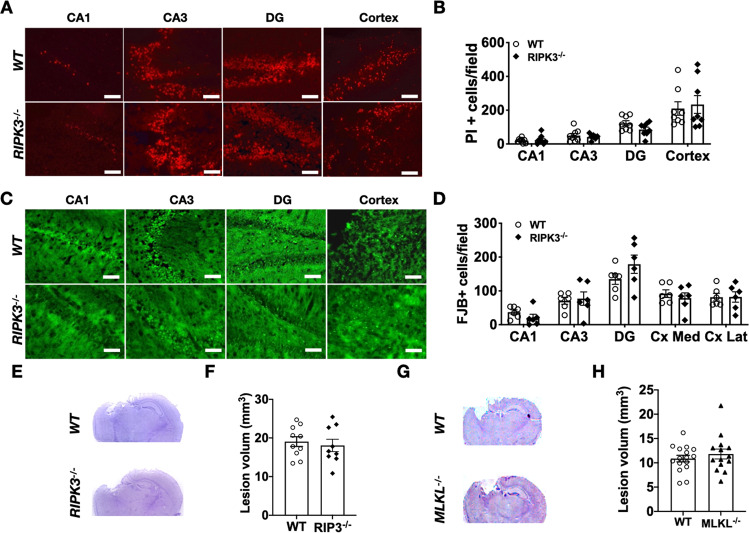
Effect of genetic inhibition of RIPK3 and MLKL on acute cell death and subacute lesion volume after CCI. **A** Representative image and **B** quantification of PI + cell counts at 6 h after CCI were not different between *RIPK3*^*−/−*^ and *WT* mice in all brain regions examined. (*n* = 6–8/group, *p* = ns, Scale bar = 100 μm). **C** Representative image and **D** quantification of Fluoro Jade B + cell counts at 6 h after CCI were not different between *RIPK3*^*−/−*^ and *WT* mice in all brain regions examined (*n* = 6–8/group, *p* = ns, Scale bar = 100 μm). At 2 months after injury, lesion volume was similar between **E**, **F** *WT* and *RIPK3*^−/−^ (*n* = 9–10/group), **G**, **H** *WT* and *MLKL*^*−/−*^ (*n* = 14–16/group) mice.

There were no differences in PI+ or fluoro-Jade B+ cell counts in injured hippocampal regions in *RIPK3*^−/−^ vs. *WT* mice (Fig. 4A–D). Likewise, there was no difference in brain tissue loss between *RIPK3*^−/−^ and *WT*, or *MLKL*^−/−^ and *WT* at 6 weeks after CCI (Fig. 4E–H). These data suggest that RIPK3 might not be a predominant mediator of cell death after CCI; alternatively, RIPK3 deficiency might promote activation of alternative death programs such as ferroptosis or apoptosis that could explain why RIPK3 KO does not seem to reduce acute neuronal death after CCI.

### RIPK3 and MLKL promote blood–brain barrier damage but not brain edema early after CCI

**Fig. 5:**
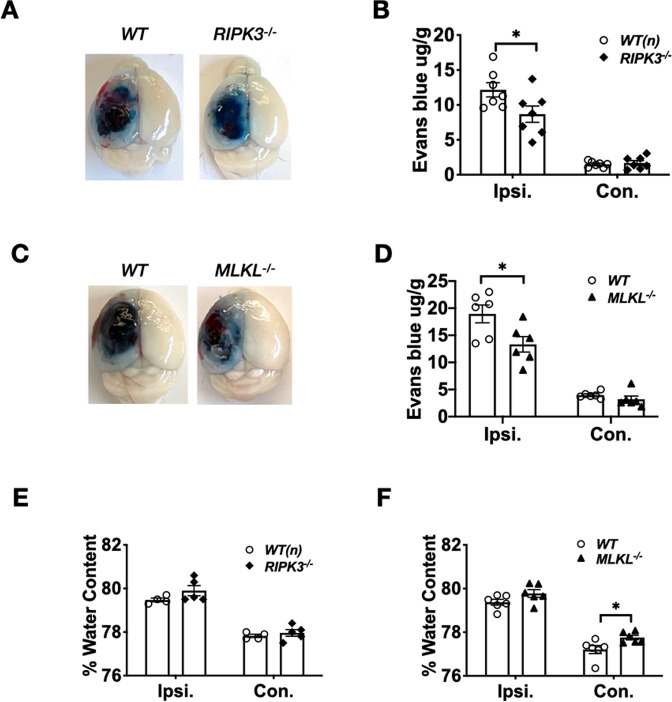
Reduced blood–brain-barrier damage in *RIPK3*^−/−^ and *MLKL*^−/−^ mice at 24 h after CCI. **A** Representative image and **B** quantification of Evans Blue extravasation in WT and *RIPK3*^−/−^. Evans blue extravasation was similar in contralateral hemispheres but decreased in ipsilateral hemispheres of *RIPK3*^−/−^ vs *WT* mice, (*n* = 7/group, *p < 0.05). **C** Representative image and **D** quantification of Evans Blue extravasation in WT and *MLKL*^−/−^. Evans Blue extravasation was similar in contralateral hemispheres but decreased in ipsilateral hemispheres of *MLKL*^−/−^ vs *WT* mice (*n* = 6/group *p < 0.05). **E**, **F** Brain water content was increased in ipsilateral vs. contralateral hemispheres in each respective group at 24 h after CCI (*p* < 0.01 for each comparison). **E** Brain water content did not differ from WT in ipsilateral or contralateral hemispheres in *RIPK3*^−/−^ mice (*n* = 4–5/group). **F** Brain water content was increased in contralateral hemispheres of *MLKL*^−/−^ vs. *WT* (*p < 0.05) but did not differ between ipsilateral hemispheres in *MLKL*^−/−^ and *WT* mice (*n* = 6/group).

At 24 h after CCI, *RIPK3*^−/−^ and *MLKL*^−/−^ mice had significantly reduced Evans blue in ipsilateral hemispheres vs. corresponding *WT* mice, while no difference was observed in contralateral hemispheres (Fig. 5A–D). CCI increased ipsilateral vs. contralateral hemispheric brain water content in all groups, but no differences in brain edema were observed among any of the groups at 24 hours after CCI (Fig. 5E, F). Notably, brain water content was greater in the contralateral hemisphere of injured *MLKL*^−/−^ vs. WT mice, but change in brain water content (ipsilateral - contralateral hemispheres) did not differ between *MLKL*^−/−^ and WT (*p* = ns, *n* = 6/group).

### Reduced brain inflammation after CCI in *RIPK3*^−/−^ mice

**Fig. 6:**
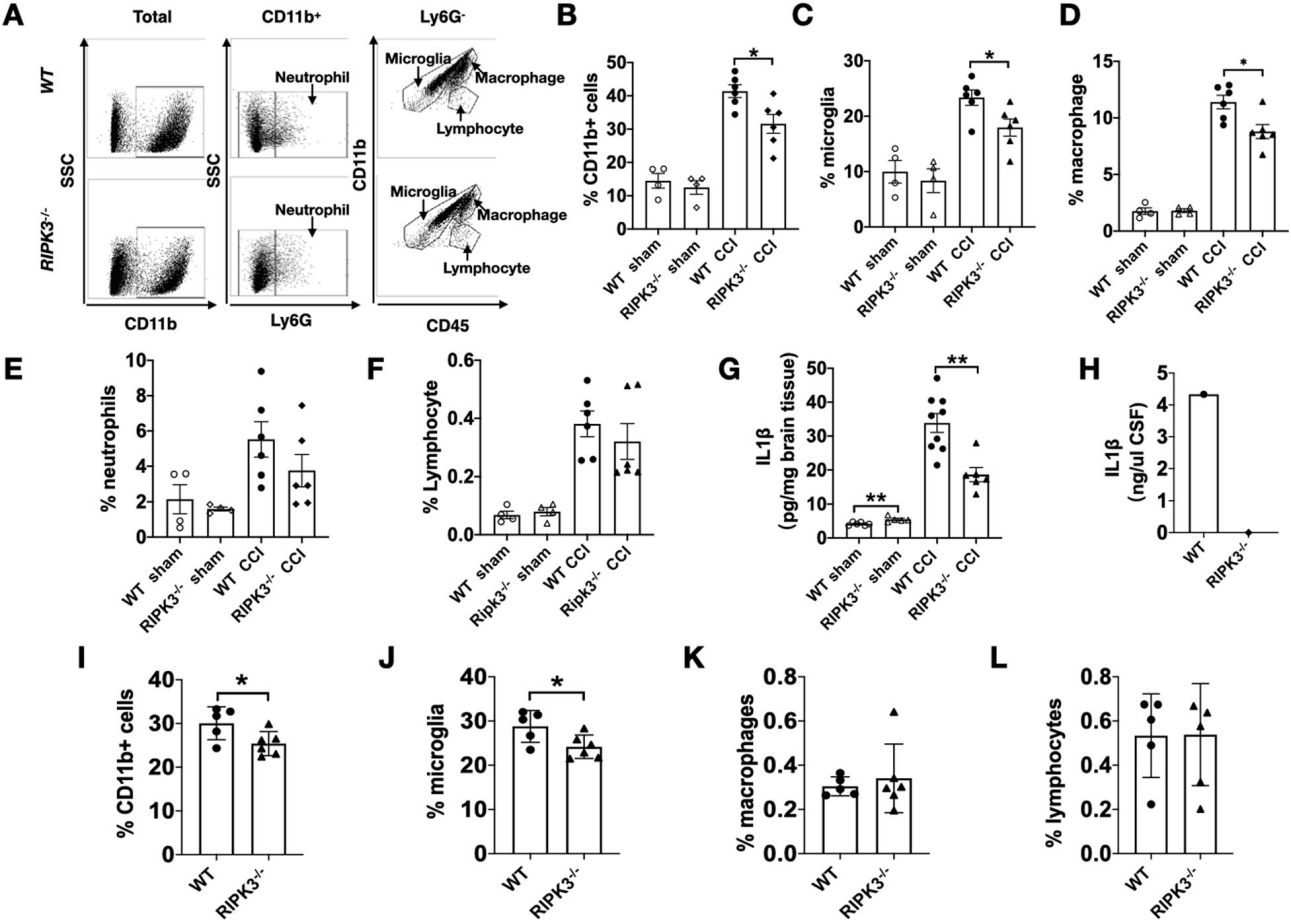
Reduced inflammation in *RIPK3*^−/−^ mice after CCI. Mice were subjected to sham or CCI and brain immune cells were assessed by FACS at 48 h using Ly6G, CD11b, and CD45 antibodies. **A**, Representative flow cytometry plots of brain cells from injured wild type and *RIPK3*^−/−^ mice. **B–D** quantitative analyses showing reduced percentages of CD11b + cells, microglia, and macrophages in ipsilateral hemispheres of *RIPK3*^−/−^ vs. *WT* mice at 48 h after CCI (*n* = 6/group, *p < 0.05). **E**, **F** No differences were observed in injured hemispheres between *RIPK3*^−/−^ and *WT* with respect to percent neutrophils and lymphocytes (*n* = 6/group). **G** Brain tissue IL-1β (measured by ELISA) was slightly increased in sham injured *RIPK3*^*-*/-^ vs *WT* indicating higher baseline IL-1β in *RIPK3*^*-*/-^(*n* = 5–6*/*group, **p < 0.01). Brain tissue IL-1β was increased by CCI in *RIPK3*^−/−^ and *WT* groups, however *RIPK3*^−/−^ mice had reduced brain tissue IL-1β at 24 h after CCI vs. *WT* (*n* = 6–9/group, **p < 0.01). **H** Decreased IL-1β in CSF (pooled from three mice per group) at 24 h after CCI in *RIPK3*^−/−^ vs. WT mice measured by ELISA. **I**–**L** At 3 weeks after CCI, injured *RIPK3*^−/−^ mice still had decreased total CD11b + cells, microglia but not in macrophage and lymphocytes in ipsilateral hemispheres (*n* = 5–6/group, *p < 0.05).

We next asked whether *RIPK3*^−/−^ mice also had less inflammatory cell infiltration into the brain because cellular inflammation may influence BBB damage and functional deficits. Cells were isolated and stained with various immune cell lineage-specific antibodies, as indicated in Fig. 6A, and analyzed by flow cytometry. Analyses of cells stained with CD11b^+^ antibodies revealed the total leukocyte population in the brain tissue harvested from *RIPK3*^*+/+*^ and *RIPK3*^−/−^mice. The frequency of CD11b^+^Ly6G^+^ neutrophils, CD11b^+^Ly6G^-^CD45^med^ microglia, and CD11b^+^Ly6G^-^CD45^high^ macrophages in the brain was similar in *RIPK3*^*+/+*^ and *RIPK3*^−/−^ sham injured mice. However, 48 h after CCI, *RIPK3*^−/−^ mice had significantly less total CD11b^+^ cells, including less macrophages and microglia in ipsilateral brain tissue compared to *RIPK3*^*+/+*^ mice but similar numbers of neutrophils and lymphocytes (Fig. 6B–F). IL-1β was reduced in ipsilateral cortex and in CSF from *RIPK3*^−/−^ vs. WT mice at 24 h after CCI (Fig. 6G, H). At 3 weeks after injury, *RIPK3*^−/−^ mice had significantly less total CD11b + cells, which were accounted for by microglia, in ipsilateral brain hemispheres (Fig. 6I–L and Supplemental Fig. 4).

### RIPK3 deletion inhibits HMGB1 release from injured brain after CCI

**Fig. 7:**
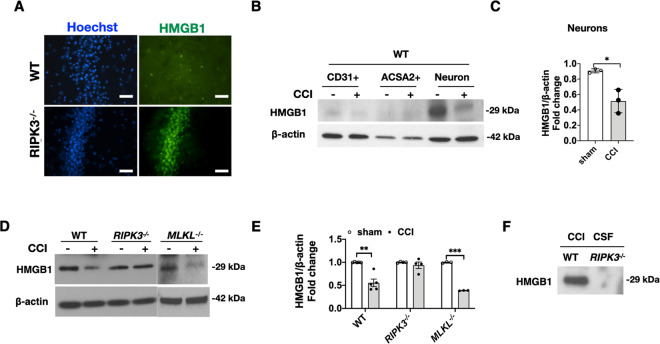
Impaired HMGB1 release after CCI in *RIPK3*^−/−^ mice. **A** Loss of immunoreactive HMBG1 in hippocampus of *WT* but not *RIPK3*^−/−^ mice at 4 h after CCI (Scale bar = 50 μm). **B** Representative western blot and **C**, densitometric quantification of immunopanned CD31 + endothelial cells, astrocytes, and neurons isolated from pericontusional tissue of sham and injured WT mouse brain at 24 h after CCI show that HMGB1 is predominantly released from neurons (*n* = 3–4/group, *p < 0.05). **D** Representative western blot and **E** densitometric quantification of HMGB1 in brain tissue homogenates showed decreased HMGB1 expression in injured brain vs. sham in WT and *MLKL*^−/−^ mice, but expression was maintained in *RIPK3*^−/−^ (*n* = 3–5/group, *p < 0.05). **F**, Western blot detection of HMGB1 in cerebrospinal fluid (CSF) from injured WT but not *RIPK3*^−/−^ mice (CSF from three mice/group combined for one experiment).

RIPK3 may drive tissue inflammation through necroptosis-associated release of damage-associated molecular patterns such as high mobility group Box-1 (HMGB1) [37], which subsequently trigger cytokine release and inflammation [11]. RIPK3 can also promote inflammation through kinase-independent mechanisms [10, 13]. HMGB1 staining in sham injured mice was nuclear whereas in CCI mice staining was either not detected in the nucleus, or cytosolic translocation of HMGB1 was detected. Immunohistochemical analysis of HMGB1 showed that CA3 neurons were a major source of HMGB1 loss early (4 h) after CCI and that HMGB1 appeared to be maintained in *RIPK3*^−/−^ CA3 neurons (Fig. 7A). Western blot analysis confirmed that neurons were the major cell type releasing HMGB1 in the CCI model (Fig. 7B). In mouse cortex, HMGB1 expression was reduced at 24 h after CCI in *WT and MLKL*^−/−^ vs. their respective shams but was maintained similar to sham in *RIPK3*^−/−^ brain tissue, indicating lack of HMGB1 release in

*RIPK3*^−/−^ mice (Fig. 7D, E). Moreover, HMGB1 was detected at 24 h after CCI in the cerebrospinal fluid (CSF) of *WT* but not *RIPK3*^−/−^ mice, again indicating decreased release of HMGB1 into the extracellular space after CCI in *RIPK3*^−/−^ mice (Fig. 7F).

## Discussion

We report the first systematic study of cell-specific expression and genetic inhibition of RIPK3 vs. MLKL in a preclinical TBI model. RIPK3 was activated in neurons, endothelium, and immune cells whereas MLKL was activated in neurons after CCI. *RIPK3*^−/−^ and *MLKL*^−/−^ mice both had reduced BBB damage, which corresponded to RIPK3 but not MLKL activation in endothelium. Improved motor and cognitive function observed in *RIPK3*^−/−^ (but not *MLKL*^−/−^) mice corresponded with reduced brain inflammatory leukocyte infiltration, HMGB1 release, and IL-1 beta activation but not reduced acute neuronal death or lesion volume. Altogether, our data suggest that RIPK3 is a major driver of outcome after cerebral contusion by mechanisms other than necroptosis [38, 8].

Necroptosis evolved in mammals and other vertebrates as an alternative to caspase-mediated apoptosis, presumably to limit the spread of viral infections [39]. Caspase-8 inhibits necroptosis by cleaving RIPK1 at Asp324 [40], and inhibition or genetic deletion of caspase-8 sensitizes cells to necroptosis [41, 42]. Caspase-8 inactivation in brain tissue promotes necroptosis in human neurodegenerative diseases [43]. Caspase-8 cleavage and enzymatic activity was reduced in ipsilateral vs. contralateral hemispheres at 24 h after CCI. These conditions would be expected to favor assembly of a necrosome complex, phosphorylation of MLKL, and induction of necroptosis [42]. The caspase-8 activity observed in the contralateral hemispheres of CCI mouse brains and in normal human brain [43] may serve to physiologically limit necroptosis. Mechanistically, the observed reduction in caspase-8 activity in the ipsilateral hemisphere may be due in part to increased expression of its negative regulator FLIP-long, that we previously reported occurs after CCI [44]. Our finding that caspase-8 activity was decreased in injured brain does not necessarily imply reduced apoptosis because the intrinsic pathway can still activate caspase-3 independent of caspase-8. Indeed, we and others have shown that caspase-3 cleavage is induced in the ipsilateral hemisphere by CCI [45, 46].

Activation of RIPK3 and MLKL in neurons did not translate into protective effects on acute neuronal death in *RIPK3*^−/−^ and *MLKL*^−/−^ mice. *RIPK3*^−/−^ did not reduce neuronal death in an ischemic stroke model [38], but *RIPK3*^−/−^ mice had reduced biochemical markers of apoptosis in a CCI model [17], whereas *MLKL*^−/−^ mice had markedly reduced acute neuronal death after ICH [30, 47]. The most likely explanation for the lack of effect of *MLKL*^−/−^ and *RIPK3*^−/−^ on acute neuronal death in the current study is redundancy of other cell death mechanisms known to operate in CCI, such as apoptosis, ferroptosis, and pyroptosis, among others [48–50].

We found robust activation of RIPK3 in endothelium in vivo as well as in human endothelium in vitro after CCI. The latter experiments support the possibility that endothelial RIPK3 activation after CCI in mice might be cell autonomous, which would be challenging to prove in vivo. Moreover, the use of human cells bridges our studies in mice to humans with cerebral contusion, albeit using a very oversimplified TBI model. Blood–brain barrier permeability was reduced in injured *RIPK3*^−/−^ and *MLKL*^−/−^ mice, suggesting functionality of endothelial RIPK3. It is possible that BBB damage is contributed by endothelial necroptosis [51], though we did not detect pMLKL in immunopanned CD31+ cells. Alternatively, RIPK3 might mediate vascular permeability after CCI independent of necroptosis [52, 53]. Reduced inflammation in *RIPK3*^−/−^ mice may also account for decreased BBB damage after CCI, but whether this may be true for *MLKL*^−/−^ remains to be investigated. Notably, reduced BBB damage in *RIPK3*^−/−^ and *MLKL*^−/−^ mice did not translate into less brain edema, presumably because mechanisms other than vasogenic edema predominate in CCI [54].

Mice deficient in RIPK3, but not MLKL, had significantly improved motor and cognitive functional outcome, including in the MWM and NORT. These data suggest that inhibition of necroptosis, modeled in *MLKL*^−/−^ mice, does not explain the functional improvements in *RIPK3*^−/−^, which are also necroptosis deficient [6]. Our findings agree with and extend those of Liu et al. [17], who reported reduced MWM deficits after CCI in *RIPK3*^−/−^ mice but were unable to distinguish between necroptosis and other functions of RIPK3 as the underlying mechanisms. Data from the current study also argue against the possible involvement of necroptosis that we previously reported in CCI using the RIPK1 inhibitor 7-Cl-O-necrostatin-1 [27]. Alternatively, cell death and necrosome/protein expression studies were performed in hippocampus and pericontusional cortex or ipsilateral hemispheres, and quantification of cell death in ipsilateral cortex or striatum could have revealed a role for RIPK3 and necroptosis in these brain regions.

Interestingly, *RIPK3*^−/−^ mice had improved behavioral outcomes despite brain tissue damage similar to wild type mice. Dissociation between brain tissue damage and functional outcome is well reported in the TBI literature [24] and can be explained in part by the likelihood that unique mechanisms drive tissue damage versus neural plasticity and recovery of function.

Inhibition of inflammation in *RIPK3*^−/−^ mice was initially attributed to blocking necroptosis [55] but RIPK3 also regulates nuclear factor kappa B and NLRP3 inflammasome activation [8]. Improved motor and cognitive recovery in injured *RIPK3*^−/−^ mice may be at least partly attributable to reduced IL-1 beta activity, as IL-1 beta antagonists improve functional outcome in TBI models [56, 57]. Pharmacological HMGB1 antagonism has also been reported to reduce neurological deficits and histopathology in experimental TBI [58], but inducible global depletion of HMGB1 prior to CCI did not improve functional outcome, edema, or BBB damage [59]. Genetic or pharmacological inhibition of RIPK3 reduces HMGB1 release in pulmonary fibrosis and subarachnoid hemorrhage models [60, 61], and reduced HMGB1 signaling could indirectly contribute to improved functional outcome in CCI because of its role in IL-1 beta production via toll-like receptor signaling [62, 63]. Though *MLKL*^−/−^mice have not been previously reported in a cerebral contusion model, MLKL may also play a role in recovery after CCI independent of necroptosis, as MLKL induced in Schwann cells after sciatic nerve crush injury promotes myelin degradation to promote nerve regeneration independent of RIPK3 [64].

Interestingly, RIPK3-mediated cytokine expression is required to promote tissue healing and repair in cutaneous wound [65] and intestinal injury [66] models, however we found no evidence for such a requirement in CCI. Further studies examining RIPK3 in the chronic phase of TBI are needed to verify safety of therapeutically targeting RIPK3 in TBI patients. This issue is particularly important because some RIPK3 kinase point mutations (e.g., D161N) and RIPK3 kinase inhibitors cause RIPK3-dependent apoptosis [20, 67], likely dependent on conformational changes in RIPK3 [38]. Further studies are needed to assess the distinct functional RIPK3 domains as therapeutic targets for TBI.

Although RIPK1 was not the main focus of our studies, it is noteworthy that at 24 h after CCI, RIPK1 was decreased by ~95% in CD11b + cells (Fig. 1I). Ubiquitination of RIPK1 followed by proteosomal degradation is one mechanism by which cells inhibit RIPK1-dependent signaling responses. Another is cleavage of RIPK1 by caspase-8. Both mechanisms could serve as potential regulatory mechanisms to promote survival of microglia and macrophages, or to direct inflammatory signaling away from a neurodegenerative RIPK1-dependent pro-inflammatory phenotype such as that observed in a subclass of microglia in ALS models [68]. These speculations deserve further investigation in CCI models.

Our study has several limitations. Transgenic mouse models are confounded by the possibility of compensatory mechanisms during development, however RIPKs and MLKL are not required for developmental cell death [39], and the mutant mouse lines used herein do not have overtly abnormal phenotypes or differences in neuronal and overall brain cell numbers at baseline vs. WT [30, 47]. To further mitigate this caveat we pretrained mice in behavior tests and compared post-injury outcomes to pre-injury baseline. Another limitation is that Western bot analyses do not define the spatial resolution of RIPK/MLKL activation or define the percentage of endothelial cells, glia, and neurons that are involved in RIPK/MLKL activation. In addition, transient phosphorylation events outside the time points examined might have been missed by our approach.

In conclusion, our data support the premise that RIPK3 is a disease driver independent of necroptosis mechanisms, and that pharmacological therapies targeting MLKL and necroptosis per se are not likely to be clinically effective for patients with cerebral contusion.

## Supplementary information

Supplemental Figure legends

supplemental figure 1

supplemental figure 2

supplemental figure 3

supplemental figure 4

## Data Availability

All data generated or analyzed during this study are included in this published article [and its supplementary information files].
