# Supplementary material for: Genetic inhibition of RIPK3 ameliorates functional outcome in controlled cortical impact independent of necroptosis
Source: Cell Death Dis. 2021 Nov 9;12(11):1064. doi: 10.1038/s41419-021-04333-z (PMC8578385; doi:10.1038/s41419-021-04333-z)
Supplement: Supplementary file 1 — Supplemental Figure legends [file 41419_2021_4333_MOESM1_ESM.docx]

**Supplemental Material**

**Genetic inhibition of RIPK3 ameliorates functional outcome in controlled cortical impact independent of necroptosis**

**Supplemental Figure 1.** RIPK1, RIPK3, and MLKL expression in normal brain and after CCI. Representative blots for antibody specificity of ***A*,** RIPK3 and MLKL , **B**, p-RIPK3, **C**, p-MLKL showing lack of positive band in the respective knockout tissue after injury. **D,** Representative blots for antibody specificity of RIPK1 in control and RIPK1 shRNA in BV2 cells. **E,** Representative flow cytometry plots of brain cells from sham and injured wild type mice and ***C***, western blot of RIPK1, RIPK3 and MLKL expression in sorted microglia and macrophages. ***G***, Lack of non-specific phosphorylation of RIPK1 and RIPK3 in cells immune-panned from naïve mouse brain (n = 2/group). Positive control is RAW macrophage cells induced to necroptosis. ***G,*** Representative Western blot of p-MLKL in isolated CD31+ cells, CD11b+ cells and neurons in WT and *RIPK3^-/-^* mice. p-MLKL is induced after CCI in all three cell populations from *WT(n)* but not *RIPK3^-/-^* mice. ***H***, Representative immunoprecipitation (IP) with RIPK3 pull down and western blot analyses for RIPK1 at 3hr and 48hr after injury showing RIPK1-RIPK3-MLKL interaction early after CCI.

**Supplemental Figure 2.** p-RIPK1 and RIPK1, p-MLKL and MLKL expression in 3D human brain endothelial cells after CCI in vitro. **A,** Representative western blots and densitometries of **B**, p-RIPK1 and RIPK1, **C**, p-MLKL and MLKL immunoactivity in 3D human brain endothelial cells at 24h showed no difference in sham and CCI groups. (n=6/group).

**Supplemental Figure 3.** Morris water maze hidden platform performance and swim speeds. Baseline MWM hidden platform performance was similar between **A,** *WT* and *RIPK3^-/-^* (n = 9-12/group), **B,** *WT* and *MLKL^-/-^* (n = 15-16/group). Platform crossings in the probe test were not difference between **C,** *WT* and *RIPK3^-/-^* (n = 9-12/group), **D,** *WT* and *MLKL^-/-^* (n = 14-15/group). Swim speed in hidden platform trials after CCI was also similar between **E,** *WT* and *RIPK3^-/-^* (n = 9 -12/group), **F,** *WT* and *MLKL^-/-^* (n = 14-15/group).

**Supplemental Figure 4.** Representative flow cytometry plots of brain cells from injured wild type and *RIPK3^-/-^* mice at 3 weeks after CCI.
