# Supplementary figures and images for: Genetic inhibition of RIPK3 ameliorates functional outcome in controlled cortical impact independent of necroptosis
Source: Cell Death Dis. 2021 Nov 9;12(11):1064. doi: 10.1038/s41419-021-04333-z (PMC8578385; doi:10.1038/s41419-021-04333-z)

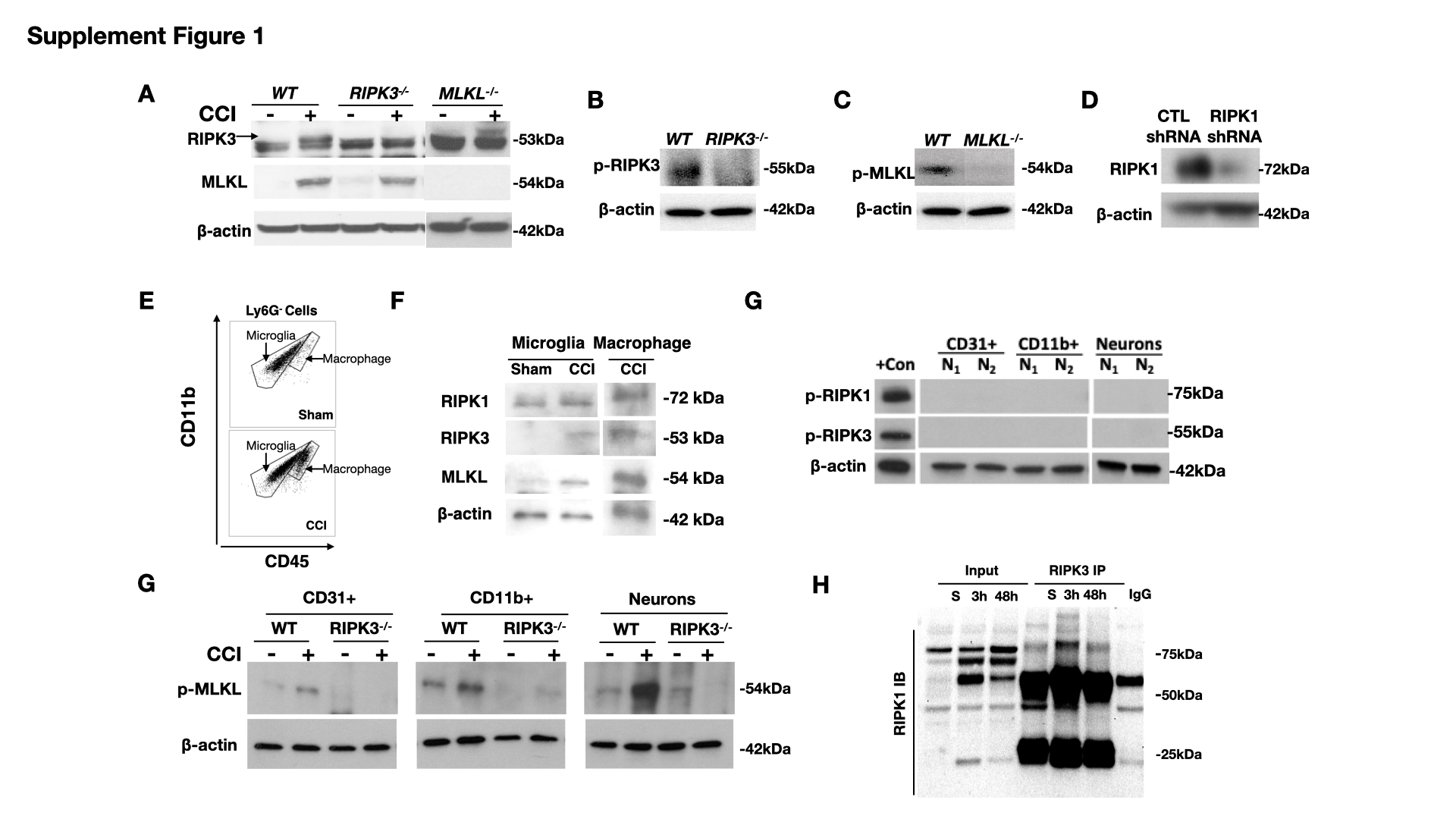

Supplement: Supplementary file 2 — supplemental figure 1 [file 41419_2021_4333_MOESM2_ESM.tif]

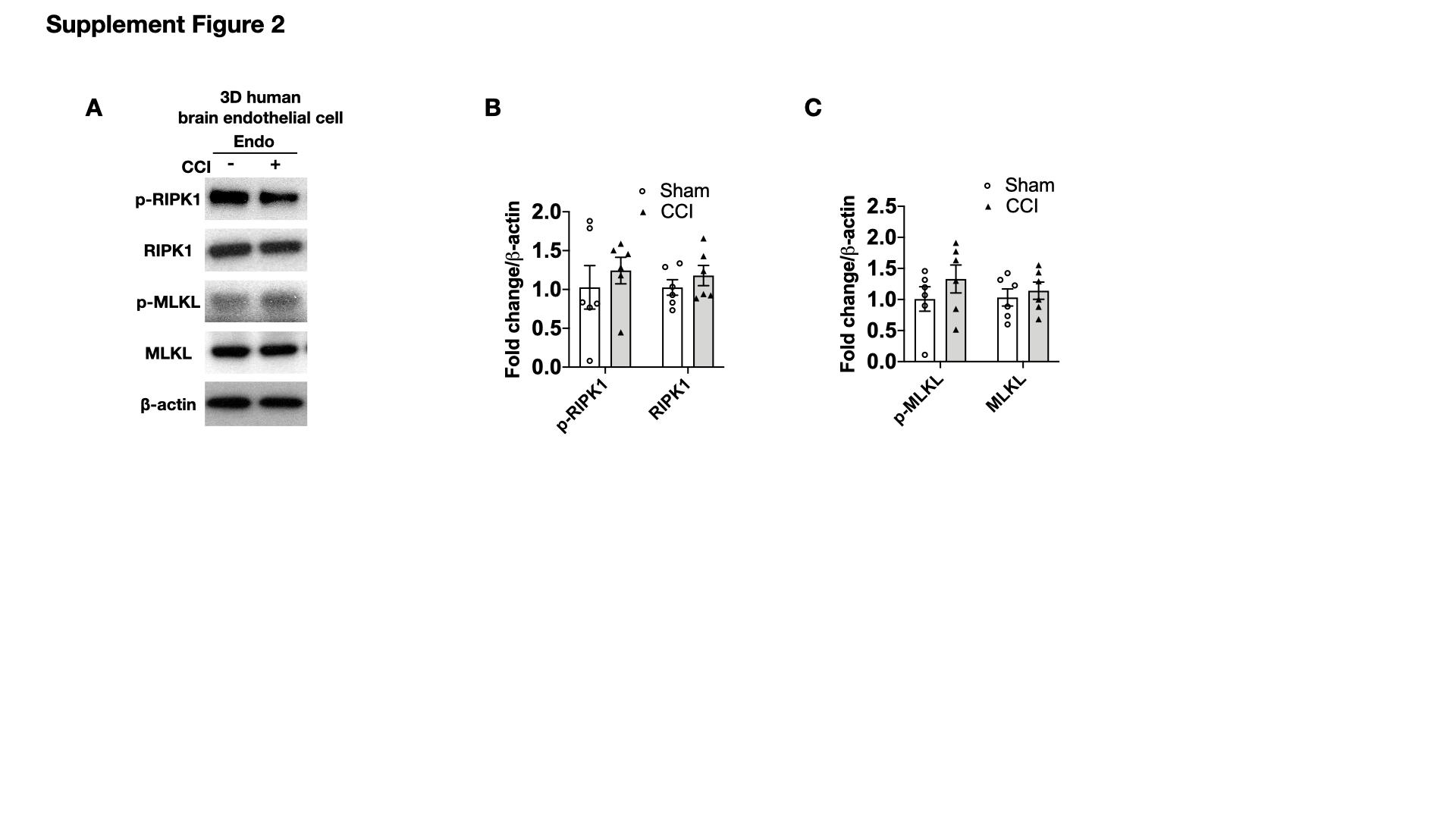

Supplement: Supplementary file 3 — supplemental figure 2 [file 41419_2021_4333_MOESM3_ESM.tif]

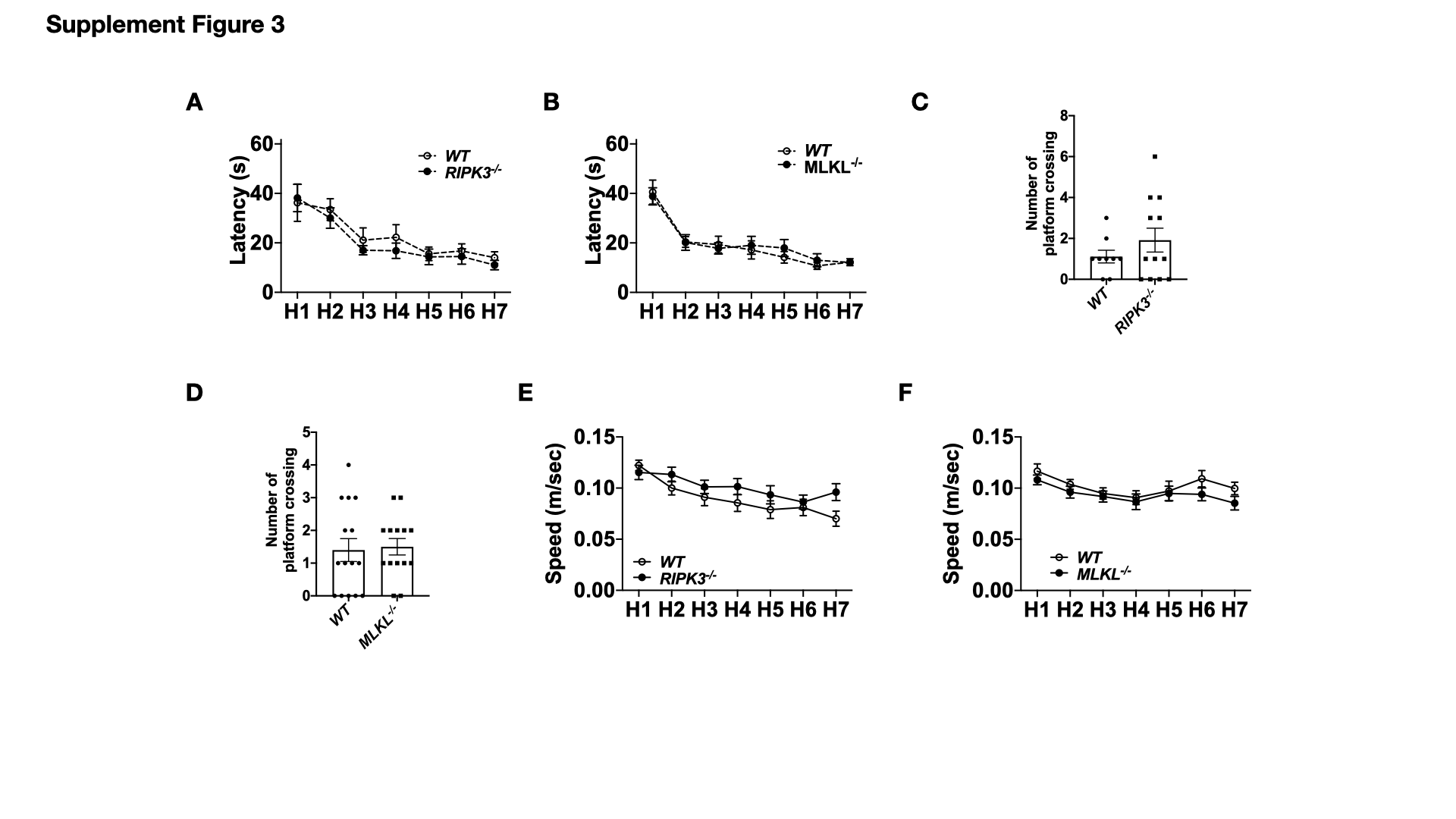

Supplement: Supplementary file 4 — supplemental figure 3 [file 41419_2021_4333_MOESM4_ESM.tif]

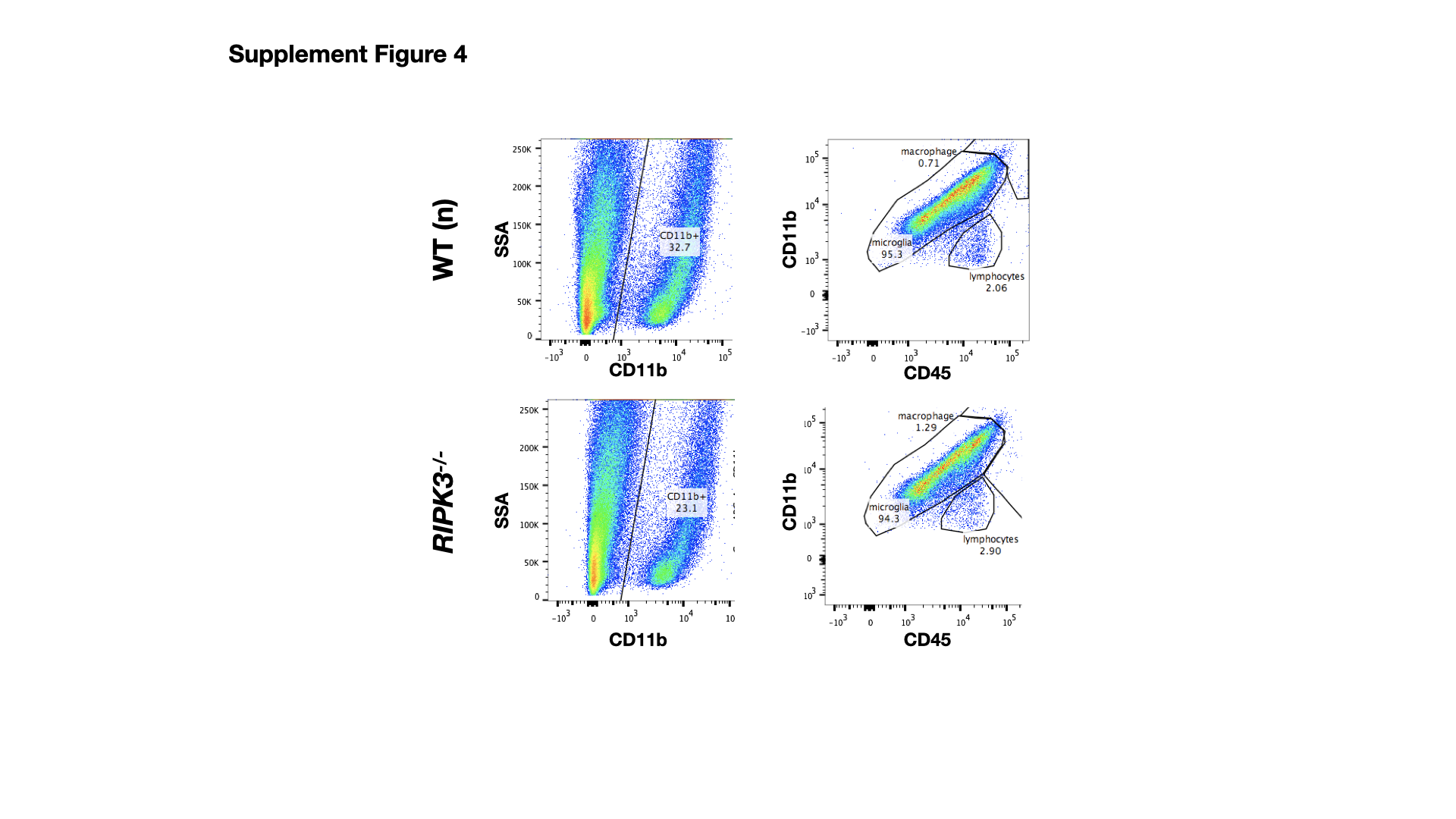

Supplement: Supplementary file 5 — supplemental figure 4 [file 41419_2021_4333_MOESM5_ESM.tif]
